# P-2121. Refining Empiric Therapy: The Influence of Risk Stratification on Antibiotic Prescribing for Diabetic Foot Infections (DFI) Utilizing an Internal Guideline

**DOI:** 10.1093/ofid/ofaf695.2285

**Published:** 2026-01-11

**Authors:** Diana Doan, Theresa Jaso, Dusten Rose, Christina Shields, Fiorella Yep Mendizabal, Alec Wesolowski

**Affiliations:** Orlando Health Arnold Palmer Hospital for Children, Winter Park, FL; Ascension Seton Medical Center Austin, Austin, Texas; bioMerieux, Austin, Texas; Ascension Seton, Austin, Texas; Ascension Seton, Austin, Texas; Dell Children's Medical Center, Austin, TX

## Abstract

**Background:**

Diabetic foot ulcers (DFUs) are common in individuals with diabetes mellitus (DM), frequently leading to infection, hospitalization, and amputation. Broad-spectrum empiric antibiotics targeting methicillin-resistant Staphylococcus aureus (MRSA) and Pseudomonas aeruginosa (PSA) are often overprescribed. In 2021, Ascension Seton implemented a local diabetic foot infection (DFI) guideline using a risk-stratified approach to optimize empiric antibiotic prescribing. This study evaluates its impact on antimicrobial stewardship and clinical outcomes.

**Methods:**

This multi-site, retrospective cohort study included adults hospitalized with DFI or diabetes-related foot osteomyelitis (DFO) between January 2017 and September 2024. Patients were excluded if treated with antibiotics for < 36 hours, had necrotizing fasciitis, or surgical site infections. The primary outcome was rate of empiric anti-MRSA and/or anti-PSA antibiotic use pre- vs. post-guideline implementation. Secondary outcomes included guideline concordance, escalation/de-escalation, treatment failure, 90-day readmission, and safety. Statistical comparisons used Chi-square and nonparametric tests.

**Results:**

Of 170 patients (pre-guideline: n=102; post-guideline: n=68), empiric anti-MRSA use decreased from 66.7% to 54.4% (p=0.113) and anti-PSA use from 69.6% to 58.8% (p=0.156). Guideline concordance improved (46.1% vs. 55.9%, p=0.084). There were no significant differences in treatment failure (37.3% vs. 35.2%, p=0.813), 90-day readmission (28.4% vs. 29.4%, p=0.583), or adverse drug events (6.9% vs. 2.9%, p=0.288). No Clostridioides difficile infections or mortality occurred in either group.

**Conclusion:**

A risk-stratified DFI guideline reduced empiric broad-spectrum antibiotic use and improved guideline adherence without compromising clinical outcomes. These findings support risk-based empiric therapy as a safe and effective antimicrobial stewardship strategy in DFI management.

**Disclosures:**

All Authors: No reported disclosures

